# Evaluation of the relationship among gene expressions and enzyme activities with antioxidant role and presenilin 1 expression in Alzheimer's disease

**DOI:** 10.1111/jcmm.17953

**Published:** 2023-09-29

**Authors:** Mesut Işık, Abdullah Tunç, Hatice Esra Duran, Muhammet Emin Naldan, Aslan Yılmaz, Mehmet Nuri Koçak, Şükrü Beydemir

**Affiliations:** ^1^ Department of Bioengineering, Faculty of Engineering Bilecik Şeyh Edebali University Bilecik Turkey; ^2^ Department of Occupational Health and Safety, Faculty of Health Sciences Bingöl University Bingöl Turkey; ^3^ Department of Medical Biochemistry, Faculty of Medicine Kafkas University Kars Turkey; ^4^ Department of Anesthesia Regional Training and Research Hospital Erzurum Turkey; ^5^ Department of Neurology Çekirge Public Hospital Bursa Turkey; ^6^ Department of Neurology, Faculty of Medicine Atatürk University Erzurum Turkey; ^7^ Department of Biochemistry, Faculty of Pharmacy Anadolu University Eskişehir Turkey

**Keywords:** Alzheimer's disease, antioxidant status, enzyme activity, gene expression, paraoxonase 1, presenilin 1

## Abstract

It is known that oxidative stress originating from reactive oxygen species plays a role in the pathogenesis of Alzheimer's disease. In this study, the role of antioxidant status associated with oxidative stress in Alzheimer's disease was investigated. Peripheral blood samples were obtained from 28 healthy individuals (as control) and 28 Alzheimer's patients who met the National Institute of Neurological and Communicative Diseases and Stroke/Alzheimer's Disease and Related Disorders Association criteria. Catalase, glutathione S‐transferase and paraoxonase 1 enzyme activities in blood plasma and glutathione S‐transferase enzyme activities in erythrocytes were determined by spectrophotometer. Catalase, glutathione S‐transferase and presenilin 1 gene expressions in leukocytes were determined using qRT‐PCR. Data were analysed with SPSS one‐way anova, a LSD post hoc test at *p* < 0.05. The activity of each enzyme was significantly reduced in Alzheimer's patients compared to control. The catalase gene expression level did not change compared to the control. Glutathione S‐transferase and presenilin 1 gene expression levels were increased compared to the control.

## 1 INTRODUCTION

In elderly people, Alzheimer's disease (AD) is the most common type of dementia. According to estimates, 47.5 million individuals worldwide suffer from dementia. Based on WHO estimates, the number of dementia patients is expected to rise to 75.6 million by 2030.^1^ AD is characterized by severe mental impairment and cognitive decline in addition to neuron loss. Its neuropathological symptoms include the development of senile plaques (SPs) and neurofibrillary tangles (NFTs), which cause synapses to break down, and finally neuronal death, which progresses with ageing in the brain. The accumulation of hyperphosphorylated tau inside neurons results in NFTs. Extracellular deposits of the amyloid β‐peptide (Aβ) make up the majority of SPs.^2^ SPs consist of Aβ‐insoluble, a peptide made up of 39 or 42 residue peptide fragments. Additionally, Aβ promotes the production of hydroxyl radicals.^3^ Aβ is derived from the amyloid precursor protein (APP). Early‐onset autosomal dominant AD results from mutations in APP and presenilin 1 (PSEN1). APP and PSEN1 mutations alter the synthesis of Aβ, which plays a central role in AD.^4^ The pathophysiology of AD is multifactorial involving vascular disorder,^5^, ^6^ endamaged mitochondrial function with over‐production of reactive oxygen species (ROS). Numerous studies show that the pathophysiology of AD includes the influence of oxidative stress.^7^, ^8^, ^9^ In a study, it was stated that the transformation of soluble amyloid into an insoluble fibril form contributed to the progression of AD caused by oxidative stress.^10^ It has been shown that oxidation of tau proteins in vitro by free radicals, characteristic of AD, can cause dimerization and polymerization of this protein.^11^

Oxidative stress is an unbalance in the redox state, relating to the constitution of excess ROS or the defect of the antioxidant system. It is made up of non‐radical species like hydrogen peroxide, free radicals like the hydroxyl radical and superoxide anion and reactive oxygen species. These free radicals are produced in the cell during regular cellular metabolism as a result of processes involving the cytochrome P450 enzyme, the β‐oxidation of fatty acids, the mitochondrial electron transport chain and the respiratory burst during the immunological defence. All cellular macromolecules, such as lipids, proteins and nucleic acids, are susceptible to oxidation at the hands of free radicals. In this respect, its breakthrough is important. Otherwise, this unbalance may result from disease stressors. They accumulate in cells and cause loss of function. Oxidative damage can be detected by measuring antioxidant enzyme activities. Several enzymes are known to have major antioxidant activity, such as catalase (CAT) and glutathione S‐transferase (GST). Two molecules of hydrogen peroxide are broken down by the high‐turnover enzyme CAT into one oxygen molecule.^12^ For this reason, it is known to have a protective role in various pathological conditions such as atherosclerosis,^13^ ageing^14^ and neurodegenerative diseases.^15^ GST are responsible for the cellular detoxification of many cellular endogenous and exogenous components. They catalyse the nucleophilic attacks of GSH on toxic compounds and organic hydroperoxides. It has a protective role against therapeutic agents and cellular oxidative stress.^16^ Potential links between oxidative stress and the pathophysiology of AD have been reported in a study. It has been stated that plasma GSH, a biomarker of oxidative stress, can be used as a potential biomarker for the development of AD and longitudinal relationships between plasma GSH and the risk of AD development. In addition, many studies have been conducted in the literature expressing the relationship of oxidative stress biomarkers CAT and paraoxonase 1 (PON1) with AD.^17^, ^18^, ^19^

Paraoxonase 1 is a high‐density lipoprotein (HDL)‐related serum enzyme in plasma which can inhibit low‐density lipoprotein (LDL) oxidation by HDL and prohibit the generation of lipoperoxide in LDL.^20^ In PON1 knockout mice, the antioxidant capacity of HDL decreased, LDL became sensitive to oxidation and its oxidation level increased.^21^ Therefore, the PON1 level may be a direct marker of HDL‐related antioxidant capacity. In addition, PON1 has an antioxidant capacity that act a part in the detoxification of xenobiotics.^22^ It has also been shown to have an effective defence against atherosclerosis. Its serious role is stroke, which is one of the neurological diseases.^23^ In recent years, many studies have shown that there is an association between oxidative stress‐related neurodegenerative disorders such as AD and PON1.^24^, ^25^ However, although it is well‐recognized that oxidative stress contributes to neurological disorders, this mechanism is not fully clear.

Although AD patients are known to be vulnerable to multiple complications connected to cognitive function, creative therapeutic strategies to reverse this progression are unfortunately insufficient. Therefore, in this study, we aimed to compare the antioxidant enzyme status and antioxidant gene expressions in patients with AD. Accordingly, CAT, GST and PON1 enzyme activities and CAT, GST and PSEN1 gene expression levels were evaluated. The oxidative stress‐based relationship between these parameters and AD was investigated.

## 2 MATERIALS AND METHODS

Consent was obtained from all patients or their families. Patients or their families' permission (AD Assessment Scale‐Cognitive [controls 7 ± 0.2; AD 18.5 ± 0.3]) was obtained before blood samples were taken. Patients with AD had MMSE minimental memory test, clinical and cranial radiological MRI‐guided Diagnostic and Statistical Manual of Mental Disorders (DSM) IV and V criteria as well as National Institute of Neurological and Communicative Diseases and Stroke/Alzheimer's Disease and Related Disorders Association (NINCDS‐ADRDA).^26^ Twenty‐eight patients with AD who submitted applications to the neurology clinic at Erzurum City Hospital and 28 healthy individuals from Turkey were selected for the study. Individuals who have a history of infection or inflammation, cancer, autoimmune disorder, haematological disorder, liver or kidney disease or use of immunosuppressive, anti‐coagulant or anti‐inflammatory drugs in the last 2 months were removed from the study. The patient and control groups' ages were not significantly different (*p* < 0.05). Demographic data about the groups are given in Table 1. All individuals gave consent to the collection of peripheral blood samples. Plasma erythrocytes and leukocytes were separated from the blood samples and stored at −80°C until studied.

**TABLE 1:** Demographic data about the groups.

Parameters	AD group (*n* = 28)	Control group (*n* = 28)
Age (years)^a^	67.33 ± 2.39	65.43 ± 2.39
Male/female	16/12	16/12
AD Assessment Scale‐Cognitive	18.5 ± 0.3	7 ± 0.2

^a^ Data are expressed as the mean ± SD.

### 2.1 Total RNA extraction

By using the osmotic lysis procedure, leukocytes were extracted from blood samples (2.5 mL in EDTA). Using the QIAamp RNA Blood Mini Kit from QIAGEN, total RNA was isolated by the manufacturer's instructions. By measuring the absorbance at 280 nm, the concentration of the acquired total RNA was evaluated. Total RNA was stored at −80°C to obtain cDNA.

### 2.2 cDNA synthesis

cDNA synthesis was performed from total RNA by the manufacturer's instructions by the SuperScript III First‐Strand Synthesis System for RT‐PCR (Invitrogen).

### 2.3 Real‐time PCR

The batch number of GST, CAT and PSEN1‐specific primers and probes designed by QIAGEN are JN137107, JN137109 and JN137110, respectively. GGTTCTCCTTCGTGCCTGT as forward primer, AGCCCTCATCCTTCACCAC as reverse primer, GCCCTCATCAACGCGGGAGA as TaqMan probe specific to GAPDH gene as the housekeeping gene was designed manually. Both the GAPDH and the target gene were tested in triplicate in this assay. For real‐time PCR, 2 μL of the generated cDNA was utilized as a template. PCR was carried out by the manufacturer's instructions. (TaqMan FastStart Probe Master Mix, Roche). To determine relative expression levels, the previously reported 2^−ΔΔCt^ method was used.^27^

### 2.4 Catalase activity

The Nelson and Kiesow (1972)^28^ approach, modified for CAT activity, is based on the absorbance measured at 240 nm for 2 min caused by the breakdown of H_2_O_2_. The results are given as enzyme units (EU) after the enzyme activity was measured using the molar extinction coefficient (ɛ = 43.6 M^−1^ cm^−1^).

### 2.5 Measurement of glutathione S‐transferase activity

The Harvey and Beutler (1982)^29^ technique was adapted to measure GST activity using 1‐chloro‐2,4‐dinitrobenzene (CDNB) as a substrate. The reaction mixture was pre‐incubated for 10 min at 20°C with 850 μL of 0.1 M phosphate buffer (pH 6.5), 20 μL of 20 mM CDNB and 50 μL of 20 mM GSH. A molar extinction value of 9.6 mM cm^−1^ was used in the spectrophotometric measurement of the GST activity, which was initiated by the addition of 50 μL of serum, at 340 nm for 3 min.

### 2.6 Measurement of paraoxonase 1 activity

Paraoxone (diethyl p‐nitrophenyl phosphate; 1 mM) was used to measure PON1 activity in a solution of 50‐mM glycine/NaOH (pH 10.5), 1‐mM CaCl_2_ and at 25°C. Based on the detection of p‐nitrophenol at 412 nm, the paraoxonase activity test was conducted. The PON1 activity, which catalyses the hydrolysis of 1‐mmol substrate at 25°C, was determined using a spectrophotometer by using molar extinction coefficient of p‐nitrophenol (ɛ = 18.29 M^−1^ cm^−1^).^30^

### 2.7 Statistical analysis

Results were expressed as mean ± SEM (*n* = 28). GraphPad Prism (Version 8.0.2) was used to statistically analyse the findings of the mRNA expression and enzyme activity. After performing the two‐tailed *t*‐test, we used Wilcoxon signed rank test with alpha = 0.05 to identify any notable variations.

## 3 RESULTS

**FIGURE 1:**
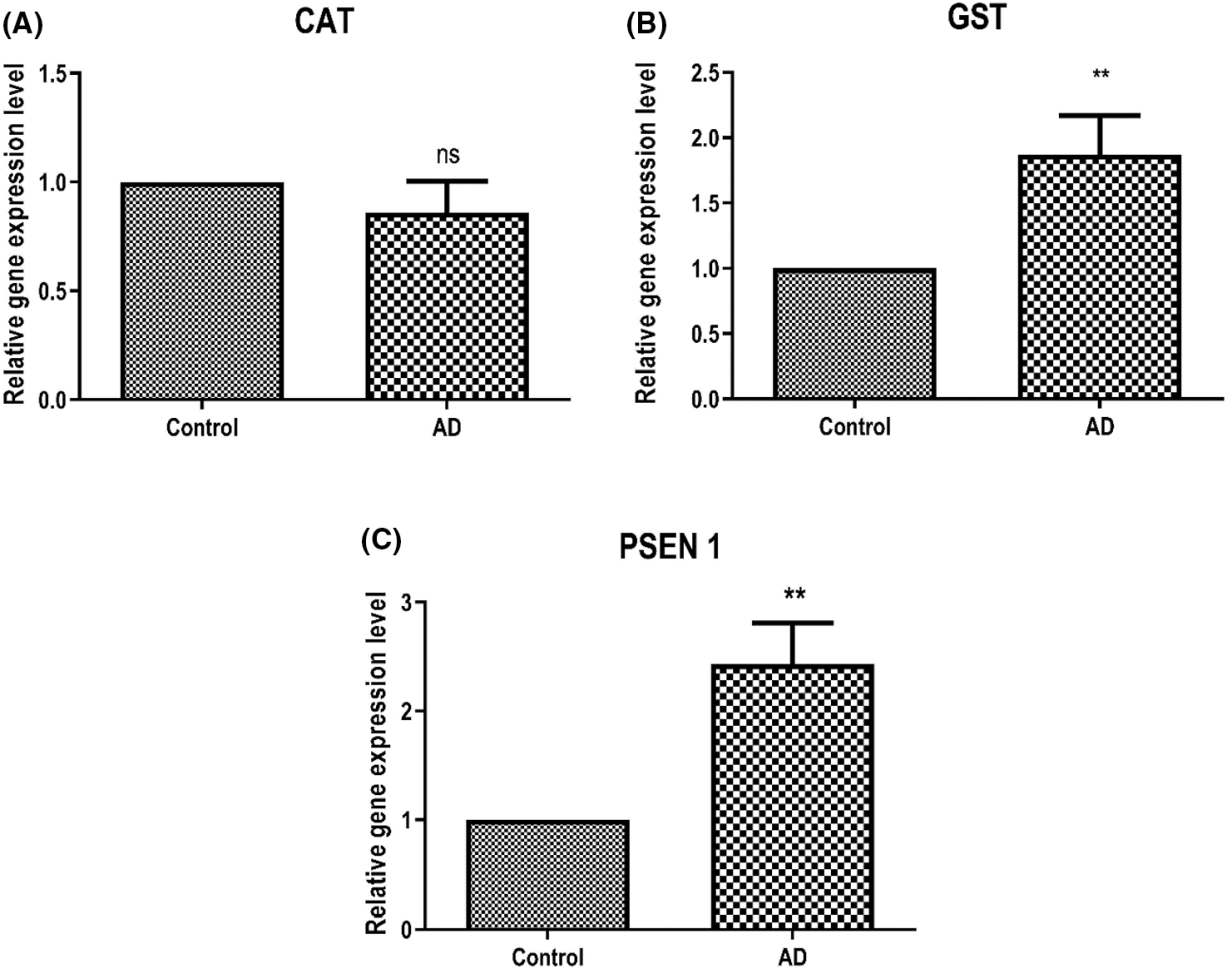
Relative expression levels of catalase (CAT; A), glutathione S‐transferase (GST; B) and presenilin 1 (PSEN1; C) compared to GAPDH in leukocytes of patients with Alzheimer's disease. The values are mean ± SEM (*n* = 28). ‘ns’ and asterisks (*) and (**) indicate ‘not significant’ and significant difference, (*p* < 0.05) and (*p* < 0.01), respectively, compared with the control group by Wilcoxon signed rank test.

Catalase gene expression levels were not found to be significant in blood leukocyte samples taken from AD compared to the control group (Figure 1A). The level of GST gene expression increased compared to the control (*p* < 0.005, Figure 1B). This increase was found to be statistically significant. The PSEN1 mRNA level was significantly increased (*p* < 0.005, Figure 1C).

**FIGURE 2:**
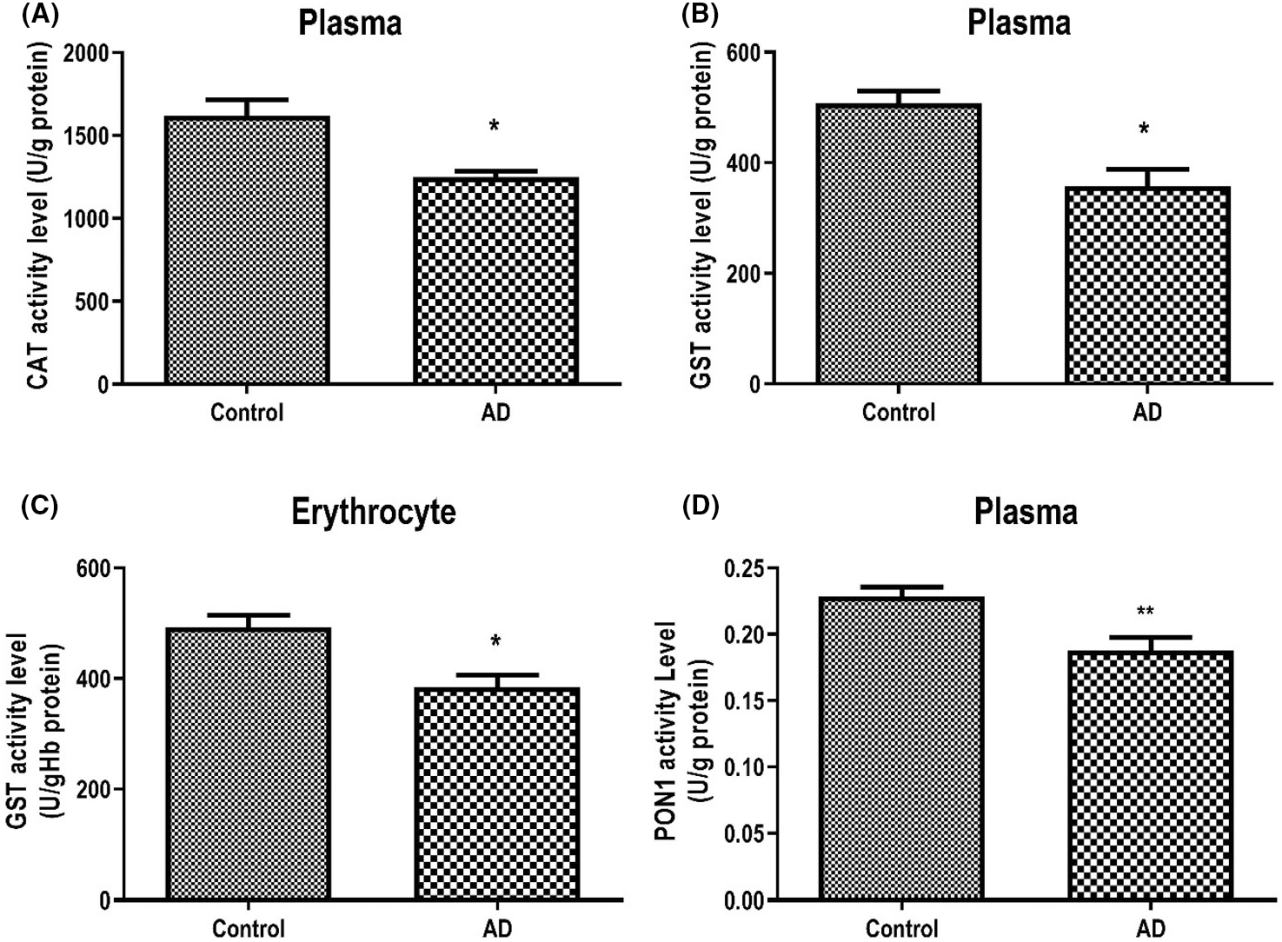
Specific activity levels of catalase (CAT; A), glutathione S‐transferase (GST; B) and paraoxonase 1 (PON1; D) in plasma and GST (C) in erythrocyte of patients with Alzheimer's disease. The values are mean ± SEM (*n* = 28). Asterisks (*) and (**) indicate significant difference (*p* < 0.05) and (*p* < 0.01), respectively, compared with the control group by Wilcoxon signed rank test.

Enzyme activities measured from plasma and erythrocytes were as follows: The change in plasma specific activity level of the CAT enzyme in AD was markedly reduced as compared to the control (*p* < 0.05, Figure 2A). Plasma GST enzyme‐specific activity level was substantially lower than the control (*p* < 0.05, Figure 2B). Erythrocyte GST enzyme‐specific activity level was decreased compared to the control, similar to plasma (*p* < 0.05, Figure 2C). Paraoxonase 1 enzyme‐specific activity in AD was lower than the control (*p* < 0.005, Figure 2D).

## 4 DISCUSSION

Işık and Beydemir (2022)^31^ demonstrated that oxidative stress occurs in AD patients, and they hypothesized that there might be a strong correlation between oxidative stress and Aβ levels. In a study conducted on aged mice, it was found that methylglyoxal, which causes oxidative damage to proteins, increases oxidative stress and causes AD‐like cognitive impairment, as well as ROS formation and increased PSEN1 gene expression. In our research, we determined that PSEN1 gene expression increased in leukocytes. Although the hypothesis of accumulation of Aβ residues is applicable, the mechanism of AD is still not fully elucidated. Studies should continue to elucidate the facts regarding the course of the neurodegenerative process. As it is known, free radicals play a significant role in this process. Numerous studies have offered proof of the negative effects of oxidative stress products on certain cellular targets in AD, even if the findings are not always consistent.^32^

Catalase is responsible for clearance through the breakdown of H_2_O_2_, so it is also necessary for cells to signal by using CAT as a signalling mechanism. In oxidative stress conditions, it is generally observed that CAT activity levels increase more than in normal conditions.^12^ It is known that Aβ causes the accumulation of H_2_O_2_ in neuronal cultures of patients with AD. It has been reported that it may lead to a decrease in enzyme activities, possibly by linking Aβ to CAT.^33^ It is hypothesized that this CAT‐Aβ interaction may have an important function in the increase of H_2_O_2_ in cells, which is linked to the aggregation of Aβ and the occurrence of oxidative stress conditions in patients with AD.^34^ It is thought that the Aβ protein directly binds to the protein and inactivates CAT activity, causing oxidative stress.^35^ Indeed, CAT catalyses half of the available hydrogen peroxide.^36^ In the previous study, the increase of PCO and the decrease of GSH in patients with AD were emphasized as indicators of the outbreak of oxidative stress. Although it increased in MDA level, no significant difference was found.^37^ In the studies, it was determined that while there was no significant change in CAT activity in the brain tissues of AD patients,^38^, ^39^ erythrocyte CAT activity showed a significant decrease.^40^ In addition, a decrease in CAT levels was noted in patients with schizophrenia, diabetes and atherosclerosis.^18^, ^34^, ^41^ In some studies, there was no change in the antioxidant enzyme levels of AD patients.^42^ It has been known for years that free radicals that cause ROS imbalance are responsible for the pathogenesis of neurodegenerative diseases.^12^, ^32^, ^43^ It has also been reported that low blood CAT levels cause oxidative stress conditions, which in turn promote type I diabetes.^44^ The relationship between the CAT enzyme and diseases has been investigated, and it has been revealed that it is associated with many diseases. More comprehensive disease‐based research worldwide is needed to fully elucidate the mechanism underlying this relationship.^35^

We observed that GST activity in plasma and erythrocytes of patients with AD decreased compared to control levels in this study. GST is known to be a group of enzymes primarily responsible for the detoxification of toxic metals.^45^ It is responsible for the detoxification of metal complexes that bind to GSH.^46^ Toxicity increases GSH concentration as it causes increased ROS as well as lipid oxidation in the body. This increase results in the activation of the glutathione transferase group of enzymes using the feedback induction state. At the same time, increased GSH activates GST transcription. This case demonstrates the role of GST in the detoxification of ROS species. Severe heavy metal exposure also revealed an inverse relationship between low GSH activity in erythrocytes and an increase in neurological disorders.^47^, ^48^, ^49^ Contrary to our study, there was no change in GST mRNA level in the AD cerebellum in another study, and it was suggested that this may be the reason for the decrease in total GST activity in the AD hippocampus.^50^ This can be caused by different tissues. In line with our study, it was determined that GST activities were decreased in the hippocampus and the amygdala of patients with AD.^51^ Indeed, inhibition of ROS requires coordinating the action of several cellular antioxidant enzymes. However, more studies are needed to better elucidate the relationship between AD and GST.

Oxidation of cholesterol‐associated HDL involved in lipid metabolism demonstrated AD sensitivity.^52^ PON1 and apolipoprotein A1 (APOA1), which is the main protein component of PON1, prevent the oxidation of LDL along with HDL.^24^ Peroxidation has an inducing effect on oxidant enzymes under normal conditions. However, the low levels of PON1 activity against this lipid peroxidation may explain pathologies such as late‐onset AD and vascular dementia.^25^, ^53^, ^54^ In addition, if the levels of polyunsaturated fatty acids are high, neurons are more damaged by free radicals.^55^ Vascular health problems can be a factor in the emergence of AD. In addition to Aβ and NFT, cerebrovascular atherosclerosis may also be a factor in the emergence of AD. Because atherosclerosis in cerebral vessels causes hypoperfusion and hypoxia, it primarily promotes the production of Aβ.^56^ In an earlier study, the PON1 level was low in cerebrovascular patients.^18^ The decreased PON1 activity in our study may indirectly indicate that it plays an important role in the pathogenesis of AD due to the risk of atherosclerosis as well as the increase in LDL oxidation.

## 5 CONCLUSION

We strengthen the prediction that there may be a link between PON1 activity and AD, although not directly. Contrary to expectations, decreased CAT activity was also associated with AD. The free radical hypothesis can be explained by the largely heterogeneous nature of AD and the fact that both genetic and non‐genetic causes are involved. There are some limitations into our study. First, the relatively small sample size may have affected the study's results and interpretation. In addition, our study did not investigate the etiopathogenic association with underlying cellular causality. Therefore, multicentre cohort studies with extensive participation are needed.

## AUTHOR CONTRIBUTIONS

**Mesut Işık:** Conceptualization (equal); data curation (equal); formal analysis (equal); investigation (equal); methodology (equal); resources (equal); validation (equal); writing – original draft (equal); writing – review and editing (equal). **Abdullah Tunç:** Data curation (equal); formal analysis (equal); investigation (equal); resources (equal); software (equal); visualization (equal); writing – original draft (equal); writing – review and editing (equal). **Hatice Esra Duran:** Resources (equal). **Muhammet Emin Naldan:** Formal analysis (equal); investigation (equal). **Aslan Yılmaz:** Investigation (equal). **Mehmet Nuri Koçak:** Resources (equal). **Şükrü Beydemir:** Conceptualization (equal); data curation (equal); formal analysis (equal); resources (equal).

## CONFLICT OF INTEREST STATEMENT

The authors have no conflicts of interest to declare.

## Data Availability

The data that support the findings of this study are available on request from the corresponding author.
